# Decreased nuclear Pten in neural stem cells contributes to deficits in neuronal maturation

**DOI:** 10.1186/s13229-020-00337-2

**Published:** 2020-06-01

**Authors:** Shin Chung Kang, Ritika Jaini, Masahiro Hitomi, Hyunpil Lee, Nick Sarn, Stetson Thacker, Charis Eng

**Affiliations:** 1 Genomic Medicine Institute, Lerner Research Institute, Cleveland Clinic, Cleveland, OH 44195 USA; 2 Cleveland Clinic Lerner College of Medicine, Case Western Reserve University, 9500 Euclid Avenue, Cleveland, OH 44195 USA; 3 Case Comprehensive Cancer Center, Case Western Reserve University School of Medicine, Cleveland, OH 44106 USA; 4 Taussig Cancer Institute, Cleveland Clinic, Cleveland, OH 44195 USA; 5 Department of Genetics and Genome Sciences, Case Western Reserve University School of Medicine, Cleveland, OH 44106 USA

**Keywords:** *PTEN* mutation, Neural stem cells, Autism spectrum disorder, Neural development, Neuronal maturation, Creb activation

## Abstract

**Background:**

*PTEN*, a syndromic autism spectrum disorder (ASD) risk gene, is mutated in approximately 10% of macrocephalic ASD cases. Despite the described genetic association between *PTEN* and ASD and ensuing studies, we continue to have a limited understanding of how *PTEN* disruption drives ASD pathogenesis and maintenance.

**Methods:**

We derived neural stem cells (NSCs) from the dentate gyrus (DG) of *Pten*^*m3m4*^ mice, a model that recapitulates PTEN-ASD phenotypes. We subsequently characterized the expression of stemness factors, proliferation, and differentiation of neurons and glia in *Pten*^*m3m4*^ NSCs using immunofluorescent and immunoblotting approaches. We also measured Creb phosphorylation by Western blot analysis and expression of Creb-regulated genes with qRT-PCR.

**Results:**

The m3m4 mutation decreases Pten localization to the nucleus and its global expression over time. *Pten*^*m3m4*^ NSCs exhibit persistent stemness characteristics associated with increased proliferation and a resistance to neuronal maturation during differentiation. Given the increased proliferation of *Pten*^*m3m4*^ NSCs, a significant increase in the population of immature neurons relative to mature neurons occurs, an approximately tenfold decrease in the ratio between the homozygous mutant and wildtype. There is an opposite pattern of differentiation in some *Pten*^*m3m4*^ glia, specifically an increase in astrocytes. These aberrant differentiation patterns associate with changes in Creb activation in *Pten*^*m3m4/m3m4*^ NSCs. We specifically observed loss of Creb phosphorylation at S133 in *Pten*^*m3m4/m3m4*^ NSCs and a subsequent decrease in expression of Creb-regulated genes important to neuronal function (i.e., Bdnf). Interestingly, Bdnf treatment is able to partially rescue the stunted neuronal maturation phenotype in *Pten*^*m3m4/m3m4*^ NSCs.

**Conclusions:**

Constitutional disruption of Pten nuclear localization with subsequent global decrease in Pten expression generates abnormal patterns of differentiation, a stunting of neuronal maturation. The propensity of Pten disruption to restrain neurons to a more progenitor-like state may be an important feature contributing to PTEN-ASD pathogenesis.

**Graphical abstract:**

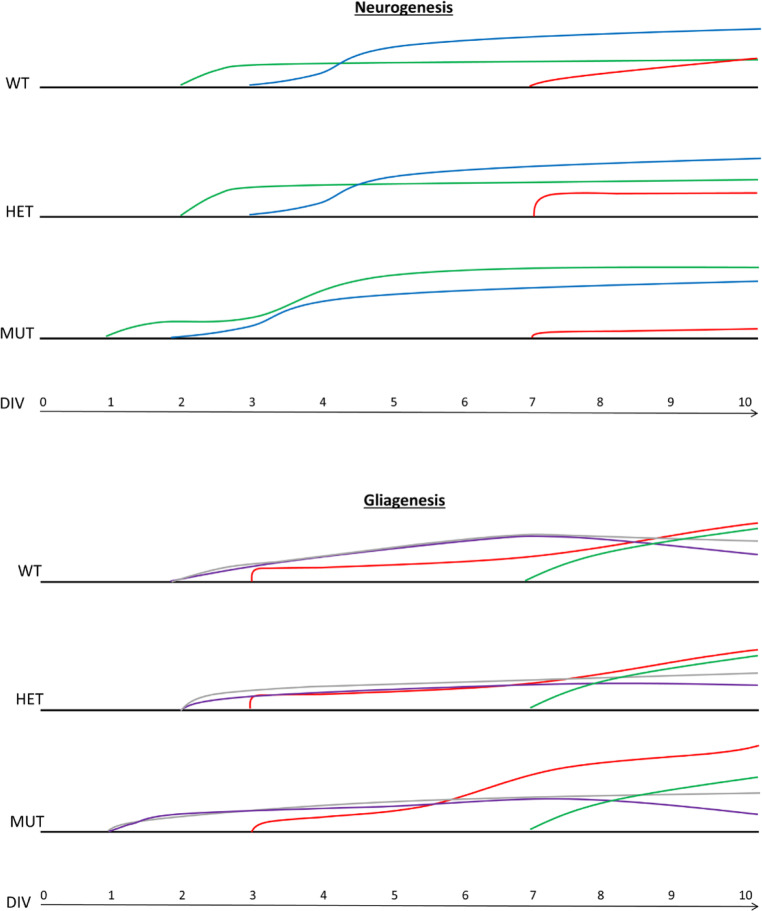

## Background

Despite the complex etiology of autism spectrum disorder (ASD), it has been established that ASD is highly heritable, where both common and rare genetic variants can contribute to the etiology [1, 2]. One of the major ASD risk genes encodes the phosphatase and tensin homolog on chromosome ten (*PTEN*) [3–5]. *PTEN* germline mutations occur in up to 10% of macrocephalic ASD, while ~ 23% of those with *PTEN* mutations will receive an ASD diagnosis [3–7]. Individuals with germline mutations in *PTEN* often have other neurodevelopmental or neurological symptoms as well: developmental delay, mental retardation, learning disability, and epilepsy [6, 7].

The strong genetic association between *PTEN* and ASD has prompted study of putative molecular mechanisms of disease. These studies have demonstrated the importance of PTEN function and the canonical dysregulation of PI3K/AKT/mTOR signaling in neuronal development, morphology, and function [8–11]. Furthermore, *Pten* is known to play a role in neurogenesis within the hippocampus. Knockout of *Pten* in neural progenitor cells (NPCs) of the hippocampus leads to a high proliferation rate and increased brain volume [12–14]. Moreover, PTEN has been shown to have synaptic functions, which are, in part, dependent on neuronal activity [15–17]. Beyond neurons, germline *PTEN* mutation has also been shown to disrupt astrocyte and oligodendrocyte development and function, indicating a PTEN-dependent complex intercellular crosstalk within the CNS during development [15, 18–22]. Despite these mechanistic insights into PTEN-mediated ASD, little is known about the impact of naturally occurring *PTEN* mutations, observed in patients and those that impact non-canonical functions (such as the increasingly salient nuclear functions of Pten), on differentiation and development of neuronal stem cells (NSCs). Moreover, ASD likely initiates in utero and/or early during development, making mechanistic insight often difficult to obtain. Therefore, NSCs derived from an established mouse model of PTEN-ASD present a useful approach for investigating ASD pathogenesis during neonatal growth [23, 24].

PTEN downstream canonical signaling typically resides in the cytoplasm. To evaluate how Pten localization and expression regulates neurogenesis and NSC differentiation, we established NSC lines from the dentate gyrus (DG) of *Pten*^*m3m4*^ mice. The *Pten*^*m3m4*^ mouse is an established model of ASD and exhibits autistic-like traits and behaviors, including sex-dependent increase in social motivation [25]. *Pten*^*m3m4*^ mutants also have deficits in motor coordination yet their motor and spatial learning and spatial and social recognition memory remain intact [25]. *Pten*^*m3m4/m3m4*^ mice present with extreme macrocephaly due to increased brain mass, which, in part, can be explained by the increase in neural soma volumes, activated astrocytes, and aberrant myelin deposits [20, 25, 26]. Follow-up studies on the *Pten*^*m3m4*^ phenotypes have demonstrated that oligodendrocyte differentiation, maturation, and myelination are aberrant and dopaminergic signaling is elevated [22, 27]. Using NSCs generated from the *Pten*^*m3m4*^ brain, we sought to understand the consequence of germline disruption of *Pten*, resulting in cytoplasmic predominant Pten expression, in influencing NSC differentiation and neuronal development and their concomitant impact on glia.

## Methods

### Isolation of neural stem cells (NSCs)

*Pten*^*wt/wt*^, *Pten*^*wt/m3m4*^, and *Pten*^*m3m4/m3m4*^ mice on a CD1 background were generated and characterized as a model of high-functioning autism as previously described [25]. All experiments were conducted in accordance with protocols approved by Cleveland Clinic’s Institutional Animal Care and Use Committee (IACUC). Mice were maintained and euthanized as previously described [24]. We adopted and modified a protocol for the isolation of neural stem cells (NSCs) from dentate gyrus (DG) for adherent cell cultures [28, 29]. NSCs were isolated from *Pten*^*wt/wt*^, *Pten*^*m3m4/m3m4*^, and *Pten*^*wt/m3m4*^ mice at postnatal day 20 (P20). Only male mice were used for isolation of wildtype NSCs, whereas a mixture of male and female mice were used for isolation of mutant NSCs. Cerebra were sectioned with a tissue slicer and stored in Hanks’ balanced salt solution (HBSS), containing 30 mM glucose, 2 mM HEPES, and 26 mM NaHCO_3_ and supplemented with Ca^2+^ and Mg^2+^. Tissues were digested into a single-cell suspension using the Neural Tissue Dissociation Kit (Miltenyi Biotec GmbH, Germany), following the manufacturer’s instructions. NSCs were obtained after a final centrifugation at 200*g* for 5 min followed by washing with N2 medium (DMEM/F12 with N2 media supplement, and l-glutamine) [28]. For adherent monolayer cell culture, cells were re-suspended in 1 mL of neurobasal growth media, 1% (v/v) Gluta-Max, B27 (without vitamin A), 2 μg/ml heparin (MilliporeSigma, Burlington, MA) in the presence of 20 ng/ml epidermal growth factor (EGF), and 10 ng/ml fibroblast growth factor 2 (FGF2) beads (StemCultures, NY) and were plated in 96-well plate coated with the 10 μg/ml Poly-l-ornithine (MilliporeSigma) and 5 μg/ml laminin. Growth media was changed every other day. All media and buffers were obtained from ThermoFisher Life Technology (Waltham, MA, USA) unless otherwise specified.

### NSC proliferation and differentiation

For random differentiation studies, NSCs were cultured in the absence of EGF and FGF2 in neurobasal media for 3, 5, 7, and 10 days. Media was replaced every other day with 50% fresh media. To promote neuronal differentiation, in certain experiments where specified, N2 media supplement was added. Otherwise, NSCs were maintained in EGF (20 ng/ml) and FGF2 (10 ng/ml) at 37 °C and 5% CO_2_. Cells were plated at a density of 1 × 10^5^ cells/ml in poly-l-ornithine/laminin coated 12-well plates in triplicate cultures. Cells were harvested, stained with 0.1% trypan blue, and counted daily for five days to obtain cell growth rate.

### Immunofluorescence

Immunocytochemistry was performed as described in Lee et al [22]. Briefly, differentiated NSCs were washed with D-PBS and fixed with 100% ice-cold methanol for 6 min. Fixed NSCs were permeabilized with 0.3% Triton-X 100/PBS for 30 min at room temperature and blocked with 10% normal goat serum (Vector, Burlingame, CA)/PBS for 1 h at room temperature. We utilized primary antibodies as NSC markers: anti-Pax6, anti-Sox2, and anti-c-Myc (Abcam, Cambridge, MA). Anti-Ki67 was used as a proliferation marker. For characterizing differentiation of immature neurons, brain-specific cell subtype markers were used, anti-Tuj1 (Biolegend, San Diego, CA) and anti-doublecortin (DCX) (Cell Signaling Technology). For mature neurons, anti-MAP2 (MilliporeSigma), anti-NeuN (MilliporeSigma), and anti-Syn (Abcam) were used, and for glial cell markers, anti-NG2 (Cell Signaling Technology, Danvers, MA), anti-Olig1 (MilliporeSigma), anti-MBP (MilliporeSigma), and anti-Gfap (ThermoFisher) were used.

### Image analysis and measurements

Images from ICC were acquired using the Leica TCS-SP8-AOBS upright confocal microscope (Leica Microsystems, GmbH, Wetzlar, Germany) at an original magnification of × 40 or × 63, or × 100. The total number of cells in each field was determined by counting DAPI-positive cell nuclei. All images are representative of 3 different areas in each coverslip, taken from at least three biological replicates. Neurite lengths were determined by tracing Tuj1+ neurons in × 40 confocal images using the Simple Neurite Tracer in Image J (FIJI, GitHub). The traces were then analyzed using Sholl Analysis to calculate the number of neurite branches in each image for more than five different areas of each slide. Images of Tuj1+ neurons were traced in more than five neurons on each image taken from three biological replicates. To obtain the ratio of Pten volume in nuclear vs cytoplasmic fractions, ICC for Pten was performed as described above. Confocal microscope images were taken at × 100 magnification, z-stacked, and processed using the Volocity® 6.3.0 program (PerkinElmer, Inc, Waltham, MA). In 3D analysis of nuclear and cytoplasmic Pten localization, the number of nuclei containing Pten as well as the total volume of Pten was calculated. All experiments were repeated at least three times in five separate locations on the slides, using 3 biological replicates.

### Western blotting

Differentiated stem cells were harvested, pelleted, and lysed in MPER buffer (ThermoFischer Scientific) with added protease inhibitor cocktails (MilliporeSigma) and incubated for 1 h on ice. Cell lysates were centrifuged for 15 min at 16,000*g* at 4 °C. Protein content was quantified using the bicinchoninic acid (BCA) assay 15–50 μg of total protein lysates were separated on a sodium dodecyl sulfate polyacrylamide gel (SDS-PAGE) and analyzed using a standard Western blot protocol. Anti-Pax6, Sox2, and c-Myc antibodies were used as neural stem markers. Nuclear and cytoplasmic Pten fractionation was performed as described [30]. Anti-Hsp90 (Cell Signaling Technology) and anti-Parp (SCT) were used as a cytoplasmic protein and a nuclear protein loading control, respectively. Anti-PTEN (Clone 6H2.1, Cascade Bioscience Inc., Winchester, MA), mTOR (CST), phosphorylated mTOR S2448 D9C2 (CST), total AKT, phosphorylated AKT S473 D9E (CST), and PI3Kinase (ThermoFisher Scientific) were used to examine the PI3K/AKT/mTOR signal pathway. For cell cycle protein assay, anti-CyclinD1, CDK4, and P27^kip1^ were used (Cell Signaling Technology). For the differentiated cells markers, we used anti-Tuj1 (BioLegend, San Diego, CA), NeuN (MilliporeSigma), and c-Fos (Cell Signaling Technology). All antibodies were used at a dilution of 1:1000 for primary and 1:2000 for secondary incubations. For the phosphorylated CREB signaling pathway assay, anti-Creb and anti-phosphorylated Creb S133 (MilliporeSigma) were used. Anti-Gapdh (Abcam) and ß-actin (ThermoFisher Scientific) were used as loading controls.

### Quantitative real-time PCR (qRT-PCR)

Total RNA from stem cells and differentiated cells was extracted using NucleoSpin® RNA Plus (Macherey-Nagel, Düren, Germany) according to the manufacturer’s instructions. RNA was reverse transcribed using a PrimeScript™ RT Reagent kit (TAKARA, Kusatsu, Shiga Prefecture, Japan). Quantitative RT-PCR was performed using the 2x Power SYBR® Green PCR Master Mix (Applied Biosystems, Foster City, CA). Real-time PCR Primers for CREB transcriptional targeted genes such as *Bdnf*, *Caln1*, and, *Neurl1b* were obtained from IDT (Coralville, IA). Relative gene expression was quantified using the comparative cycle threshold (C_T_) method using *Gapdh* as the housekeeping gene. The following primers were used: Gapdh (custom synthesis, Thermofisher): F-GGGCTGCCATTTGCAGTGGCAA, R-GTGAGTGGAGTCATACTGGAAC, Neurl1b (Assay ID # Mm.PT.58.43949047), BNDF (Assay ID # Mm.PT.58.8157970), and Caln1 (Assay ID # Mm.PT.58.7756375); predesigned qRT-PCR primers were obtained from IDT.

### Bdnf rescue of immature neurons

NSCs grown on Geltrex (growth factor depleted form obtained from Gibco, Waltham, MA) coated coverslips were induced to differentiate by growth factor deprivation in the presence or absence of BDNF (100 ng/ml) for 7 days. Cells were fixed with 3.7% (w/v) paraformaldehyde, permeabilized, and blocked with 0.1% Triton X100, 2% normal donkey serum in PBS. Fixed cells were immunostained with rabbit anti-Tuj1, mouse anti-NeuN, and rat anti-GFAP antibodies followed by incubation with donkey anti-rabbit, anti-mouse, and anti-rat IgG antibodies labeled with Alexa488, Cy3, and Cy5, respectively. DNA was stained with Hoechst 33342 (100 ng/ml).

After images of each fluorochrome were collected with a fluorescent microscope (Leica DM5000B equipped with a Leica DFC310FX digital camera), each images were processed using an image processing software, Fiji (https://imagej.net/ImageJ). After correcting background signals and shading of illumination of the images accordingly, pixel values of each fluorescence signal were integrated for individual nuclear regions. Signal from dead cells remaining on the plate was eliminated from analysis using graphical mask data region function of KaleidaGraph software (version 4.1, Synergy Software) based on footprint size and intensity of nuclear regions defined by DNA staining. Staining intensities of Tuj1 and NeuN of nuclei were compared between the cultures with or without BDNF by unpaired Wilcoxon-Mann-Whitney rank sum test using KaleidaGraph.

### Statistics

Data are expressed as the mean ± SD (standard deviation). Student’s *t* tests and one-way ANOVAs were performed, where appropriate, to compare between and among groups, respectively. All Student’s *t* tests were two-tailed and unpaired. Two-way ANOVAs were also employed where appropriate. Tukey-Kramer post hoc testing was used to compare groups and correct for multiple testing following ANOVAs. We also performed Kruskal-Wallis testing, nonparametric ANOVAs, where appropriate with Dunn’s multiple comparison post hoc testing. We considered all *p* values below 0.05 statistically significant. Statistical analyses were performed in GraphPad Prism 8 or KaleidaGraph 4.1.

## Results

### Persistence of stemness in NSCs with *Pten*^*m3m4/m3m4*^ mutations

**Fig. 1:**
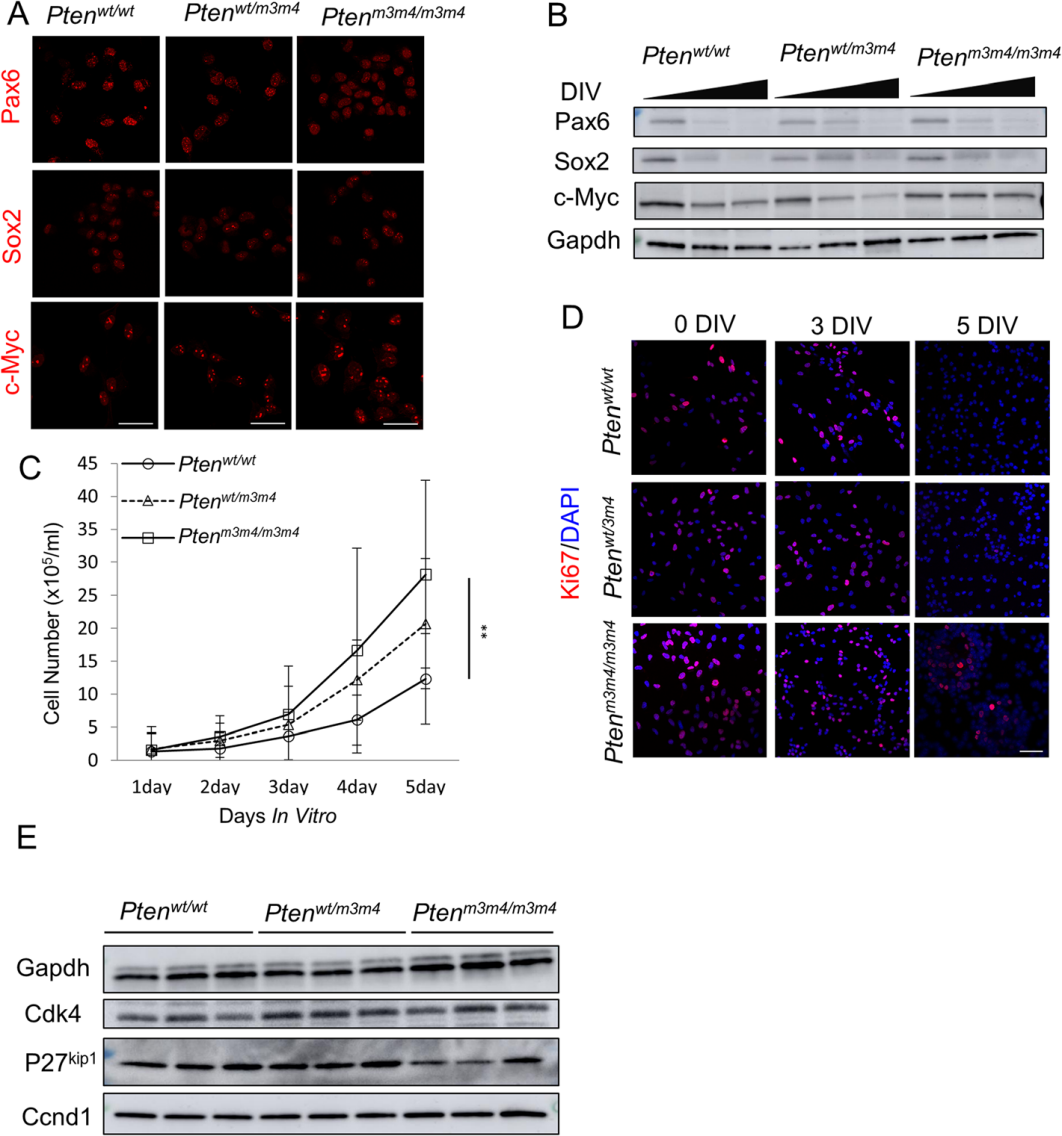
Characterized neural stem cells (NSCs) derived from *Pten*^*m3m4/m3m4*^ mice have shown slow differentiation. **a** Immunofluorescence staining for stemness makers Pax6 (top panel), Sox2 (middle panel), and c-Myc (bottom panel) at 1 day in culture with growth factors. **b** Western blots for stemness markers Pax6, Sox2, and c-Myc over 5 days of random differentiation in the absence of growth factors. Representative blot (*n* = 3). **c** Cell counting assay to assess proliferation (*n* = 5). *Pten*^*m3m4/m3m4*^ NSCs show increased proliferation at 5 DIV compared to wildtype as assessed by Kruskal-Wallis testing with Dunn’s multiple testing comparison (*p* value = 0.0012). **d** Immunofluorescence for Ki67 expression at 0, 3, and 5 DIV for all NSC genotypes. **e** Western blot analysis of undifferentiated NSCs (*n* = 3) for cell cycle progression markers (*p value < 0.05; **p value < 0.01; ****p value < 0.0001)

*Pten*^*m3m4*^ mutant neural stem cells (NSCs) cultured from the P20 dentate gyrus showed no gross morphological differences in culture compared to those derived from *Pten*^*wt/wt*^ (Additional file 1: Figure S1A). NSCs from all *Pten* genotypes expressed markers of stemness, namely, Pax6, Sox2, and c-Myc. However, expression of stem cell markers was observed to be elevated in *Pten*^*m3m4/m3m4*^ NSCs, specifically c-Myc (*p* value = 0.051; Fig. 1a; Additional file 1: Figure S1B). Overexpression of c-Myc is maintained without any decrease until at least 5 days after removal of growth factor (Fig. 1b), in contrast to *Pten*^*wt/wt*^ NSCs where c-Myc expression declines beginning at day 3 after growth factor removal (*p* value = 0.035; Additional file 1: Figure S1C). Due to this observed persistence of stemness markers in *Pten*^*m3m4/m3m4*^ NSCs, we evaluated proliferation as a consequence of persistent stemness. *Pten*^*m3m4/m3m4*^ NSCs exhibit higher proliferation rates compared to wildtype NSCs at 5 DIV (*p* value = 0.0012; Fig. 1c). Additionally, Ki67 staining was significantly increased in *Pten*^*m3m4/m3m4*^ relative to wildtype NSCs at 0 DIV (days in vitro, *p* value = 0.042) and increased in *Pten*^*wt/m3m4*^ and *Pten*^*m3m4/m3m4*^ relative to wildtype NSCs at 3 DIV (*p* value = 0.0039; *p* value < 0.0001, respectively; Fig. 1d; Additional file 1: Figure S1D). However, at 5 DIV, persistent Ki67 staining was observed only in *Pten*^*m3m4/m3m4*^ NSCs with no apparent Ki67 expression in *Pten*^*wt/m3m4*^ or wildtype NSCs (Fig. 1d). These findings on the prolonged stemness of NSCs derived from *Pten*^*m3m4*^ mutants were further supported by observing increased Ccnd1 expression (*p* value = 0.014) and decreased p27^kip1^ expression (*p* value = 0.039) in *Pten*^*m3m4/m3m4*^ NSCs (Fig. 1e; Additional file 1: Figure S1E).

### Nuclear Pten depletion in *Pten*^*m3m4/m3m4*^ NSCs

**Fig. 2:**
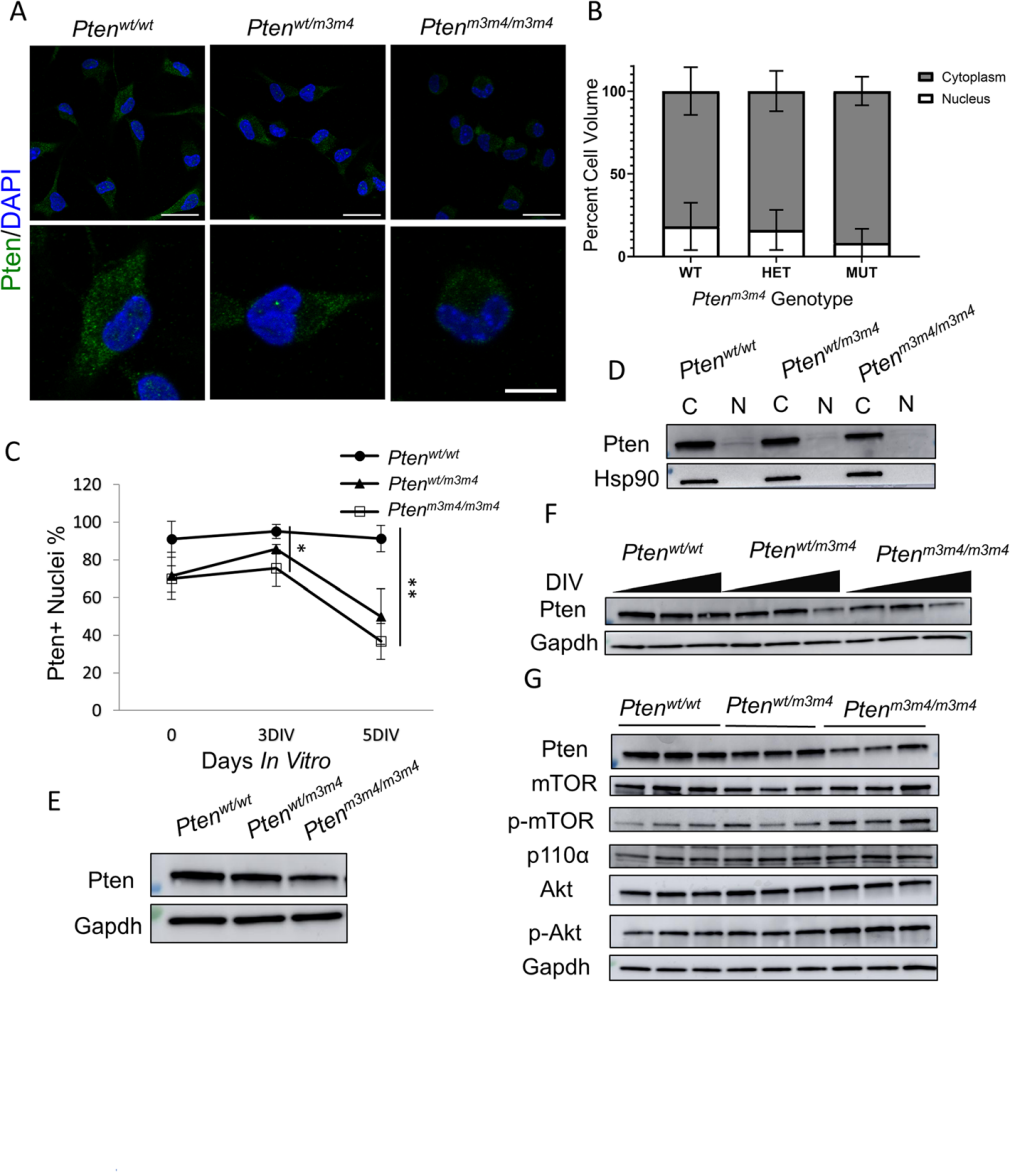
NSCs with *Pten*^*m3m4*^ mutations show decreased nuclear and global Pten levels. **a** Representative immunofluorescence staining for Pten at 1 DIV (*n* = 3). Top panel is low magnification (scale bar 25 μm). Bottom panel is high magnification (scale bar 10 μm). **b** Volocity software-assisted quantification of Pten immunofluorescence, calculating the volume of Pten protein (μm^3^) in different NSCs compartments at 1 DIV (*n* = 3). Volumes were converted to percentages of the whole. **c** Quantification of Pten immunofluorescence staining, showing percentage of Pten-positive nuclei from Pten^wt/wt^ (*n* = 4), *Pten*^*wt/m3m4*^ (*n* = 4), and *Pten*^*m3m4/m3m4*^ (*n* = 3) NSCs at 0, 3, and 5 DIV in the absence of growth factor. Kruskal-Wallis testing, accompanied by Dunn’s multiple comparison post hoc testing, found a significant difference between wildtype and *Pten*^*m3m4/m3m4*^ NSCs at 3 (*p* value = 0.035) and 5 DIV (*p* value = 0.0051). **d** Representative Western blot analysis for Pten in nuclear and cytoplasmic fractions derived from NSCs. Hsp90 was used as a cytoplasmic protein marker. **e** Representative Western blot analysis of total Pten expression in *Pten*^*m3m4*^ NSCs (*n* = 3) after 1 DIV. **f** Representative Western blot analysis of total Pten expression in *Pten*^*m3m4*^ NSCs at three timepoints over 5 days of random differentiation compared (*n* = 3). **g** Representative Western blot analysis of Pten, p110α (Pi3k), p-mTOR, and p-Akt in *Pten*^*m3m4*^ NSCs (*N* = 3) (*p value < 0.05; **p value < 0.01; ****p value < 0.0001)

*Pten*^*m3m4*^ mutation is designed to disrupt the nuclear localization-like signals of Pten, leading to predominantly cytoplasmic localization and nuclear depletion of the protein systemically in an adult mouse. In order to check if nuclear depletion of Pten was evident in NSCs, we stained for Pten to evaluate its expression and localization. We observed a decrease in expression and depletion of Pten from the nucleus in mutants (Fig. 2a). Quantification of nuclear and cytoplasmic Pten expression shows an approximately 10% reduction in homozygous mutant NSCs (*n* = 5) with a 2.5-fold reduction in the nuclear to cytoplasmic ratio relative to wildtype (Fig. 2b). As random differentiation proceeds, the number of nuclei with Pten expression significantly decreased in *Pten*^*m3m4/m3m4*^ NSCs relative to wildtype NSCs. Quantification of Pten-positive nuclei at 3 days of differentiation showed a decreased number of Pten+ nuclei in mutant NSCs compared to *Pten*^*wt/wt*^ NSCs (95% in *Pten*^*wt/wt*^ > 85% in *Pten*^*wt/m3m4*^ > 76% in *Pten*^*m3m4/m3m4*^; *p* value = 0.034). Similarly, quantification of Pten-positive nuclei at 5 days of differentiation showed decreased number of Pten+ nuclei in mutant NSCs compared to *Pten*^*wt/wt*^ NSCs (91% in *Pten*^*wt/wt*^ > 50% in *Pten*^*wt/m3m4*^ > 37% in *Pten*^*m3m4/m3m4*^; *p* value = 0.0051). The difference in Pten-positive nuclei between wildtype and *Pten*^*m3m4/m3m4*^ NSCs increases from 19 to 54% from day 3 to day 5 of differentiation (Fig. 2c). By visual inspection, the decrease in nuclear Pten expression was further confirmed by Western blot analysis after subcellular fractionation (Fig. 2d). Not only was there a decrease in nuclear Pten expression relative to cytoplasmic expression, but also a global decrease in Pten expression, which we demonstrated by Western blot analysis (*p* value = 0.0042; Fig. 2e; Additional file 1: Figure S2A). Moreover, the decline in Pten expression was further exaggerated in Pten mutant NSCs as they differentiate, where Pten expression progressively declined over 5 DIV (*p* value_DIV_ = 0.036) while also showing differences between homozygous mutant and wildtype groups (*p* value_Pten_ = 0.021; Fig. 2f; Additional file 1: Figure S2B). Given the change in Pten expression and localization, we measured changes in downstream Pi3k/Akt/mTor signaling by Western blot. Pten was again significantly decreased (*p* value = 0.034). As expected, we found significant increases in the phosphorylation of Akt, and mTor in *Pten*^*m3m4/m3m4*^ NSCs, reflecting the functional disruption of Pten’s lipid phosphatase activity in mutant NSCs (*p* values = 0.034; Fig. 2g). Together, these data illustrate that the *Pten*^*m3m4*^ mutation results in decreased Pten nuclear localization and decreased Pten overall expression and leads to dysregulation in downstream canonical signaling in NSCs. This decrease in Pten expression and its functional impact on dysregulation of downstream canonical signaling is further exacerbated as these NSCs differentiate.

### Deficits in neuronal maturation

**Fig. 3:**
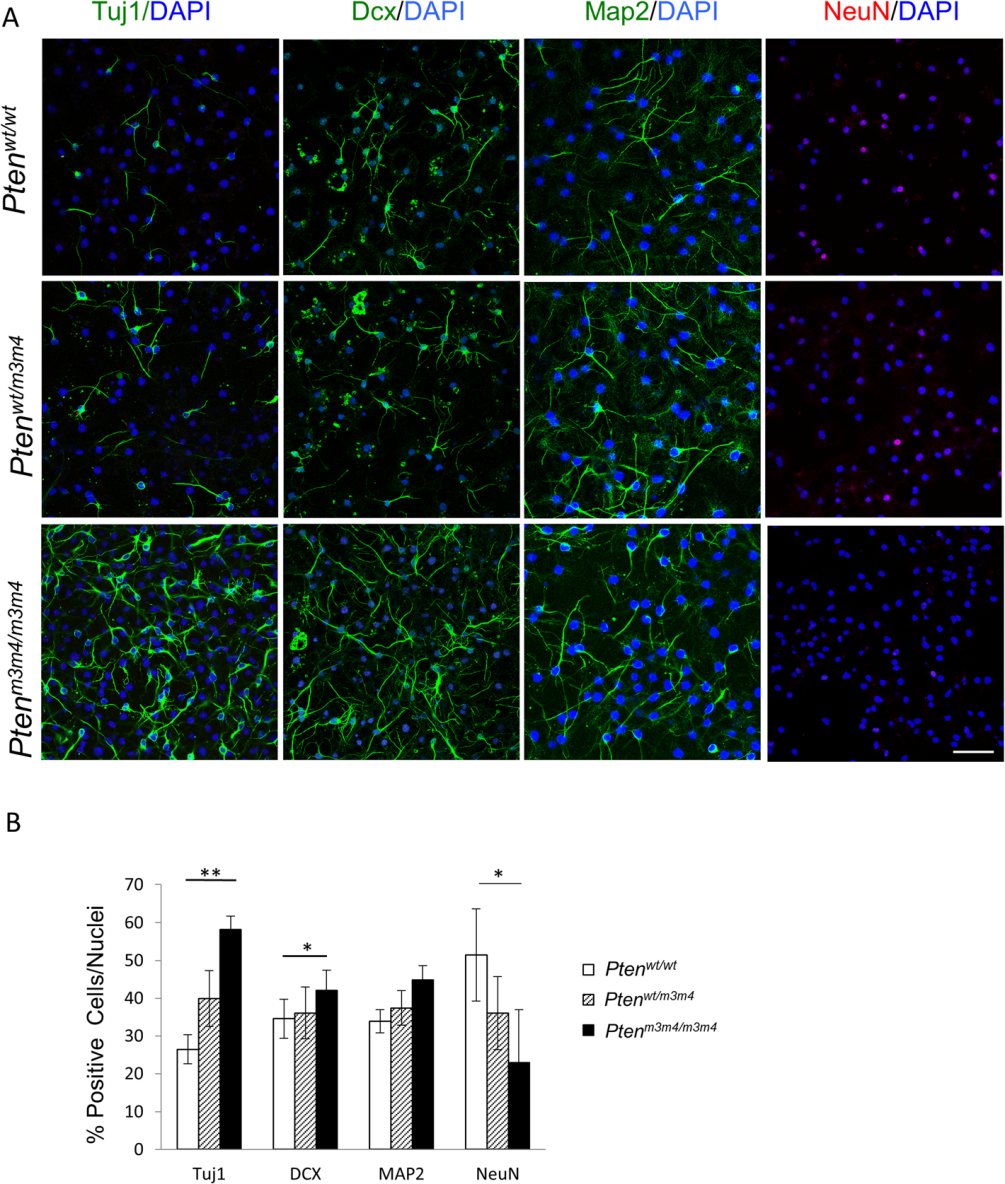
*Pten*^*m3m4*^ NSCs differentiation halts after becoming immature neurons. **a** Representative immunofluorescence staining for neuronal markers Tuj1 (green, *n* = 5), Dcx (green, *n* = 3), Map2 (green, *n* = 3), and NeuN (red, *n* = 3), showing increased presence of immature neurons in *Pten*^*m3m4/m3m4*^ NSCs compared to wildtype NSCs when cultured in the absence of specific lineage-driving growth factors for 10 days. NeuN (mature neuron marker) expression is significantly lower in *Pten*^*m3m4/m3m4*^ NSCs compared to their wildtype counterparts. **b** Quantification of immunofluorescence staining of cells expressing neuronal markers (Tuj1, Dcx, Map2, and NeuN) normalized to total number of nuclei (DAPI) per frame. Kruskal-Wallis testing, accompanied by Dunn’s multiple comparison testing, finding a significant increase in Tuj1 (*p* value = 0.0051) and Dcx (*p* value = 0.033) and a significant decrease in NeuN (*p* value = 0.015) comparing *Pten*^*m3m4/m3m4*^ and wildtype NSCs (*p value < 0.05; **p value < 0.01)

Given the changes in Pten localization and expression in concert with the persistent stemness of mutant NSCs, we decided to monitor the neuronal differentiation of *Pten*^*m3m4*^ NSCs. Thus, we allowed for 5 days of random differentiation and then stained for markers of immature (Tuj1 and Dcx) and mature (Map2 and NeuN) neurons. We found a significant increase in the numbers of Tuj1-positive (*p* value = 0.0051) and Dcx-positive (*p* value = 0.033) immature neurons in Pten^m3m4/m3m4^ NSC cultures. In contrast, expression of the mature neuronal marker NeuN was significantly decreased in differentiated *Pten*^*m3m4/m3m4*^ NSCs (*p* value = 0.015). No change was observed in expression of Map2 between any of the genotypes. Interestingly, a significantly higher number of Tuj1-positive neurons were present among differentiated *Pten*^*m3m4/m3m4*^ NSCs compared to the wildtype NSC cultures (Fig. 3a, b). These findings indicate that *Pten*^*m3m4/m3m4*^ NSCs differentiate into immature neurons earlier and more aggressively than their wildtype counterparts but subsequently delay maturation into NeuN-expressing neurons, which may be a function of the prolonged stemness of mutant NSCs.

To underscore that the observation of reduced NeuN expression in the mutant NSCs was indicative of stunted maturation, we stained for the pan-presynaptic marker synaptophysin at 7 and 10 DIV across all genotypes (Additional file 1: Figure S3B). We found that Syn expression significantly increased between 7 and 10 DIV in wildtype NSCs (*p* value = 0.034) but not in heterozygous or homozygous mutant NSCs. However, we did not find significant differences in Syn expression normalized to cell number among different genotypes (Additional file 1: Figure S3B). Although the pattern of Syn expression does not clearly show deficits in synapse formation in the mutants, it does show a lack of developmental increase in synapse formation from 7 to 10 DIV in culture.

**Fig. 4:**
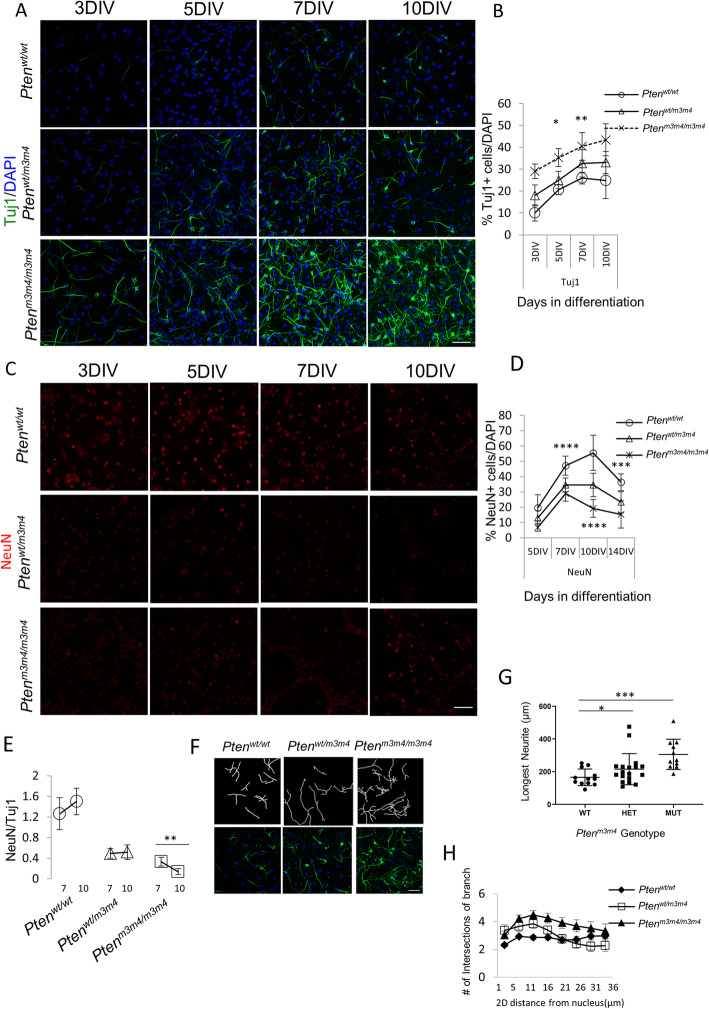
*Pten*^*m3m4*^ NSC differentiation is precocious yet abortive, leading to increased immature neuron population. **a** Representative immunofluorescence staining defines immature neuron population (Tuj1+ cell in green: DAPI+ nuclei in blue) at 3, 5, 7, and 10 days in vitro in the absence of growth factors. **b** Quantification of staining in **a**, where a significant increase in Tuj1+ neurons at 5 DIV (*p* value = 0.05) and 7 DIV (*p* value = 0.0013) comparing *Pten*^*m3m4/m3m4*^ to *Pten*^*wt/wt*^ as assessed by Kruskal-Wallis testing with Dunn’s multiple comparison testing. **c** Immunofluorescence staining for mature neuron (NeuN+ cells in red: DAPI+ nuclei in blue) population in culture for 5, 7, 10, and 14 DIV in the absence of growth factor. **d** Quantification of mature neurons in **c** (NeuN+ cells). Kruskal-Wallis testing with Dunn’s multiple comparison testing found significant decreases in NeuN+ cells at 7 (*p* value = < 0.0001), 10 (*p* value = < 0.0001), and 14 DIV (*p* value = 0.003) between *Pten*^*m3m4/m3m4*^ and wildtype NSCs and significant decreases in NeuN+ cells at 7 (*p* value = 0.018) and 14 DIV (*p* value = 0.029) between *Pten*^*wt/m3m4*^ and wildtype NSCs. **e** NeuN+ cells to Tuj1+ cells at 7 and 10 DIV (*n* = 3). Mann Whitney *U* testing found a significant different between the 7 and 10 DIV NeuN+/Tuj1+ ratios in *Pten*^*m3m4/m3m4*^ NSCs (*p* value = 0.0012). **f** Tuj1+ neurites showing arbored branched intersections of neurites (*n* = 3). **g** Simple neurite tracing quantification by Image J (Sholl analysis), showing increased mean length of longest NSC neurite in Tuj1+ cells derived from *Pten*^*m3m4/m3m4*^ NSCs (means wt vs het vs mut: 165.4 μm vs 215.5 μm vs 305.7 μm), indicating a significant increase between *Pten*^*m3m4/m3m4*^ and wildtype NSCs (*p* value = 0.0006) and between *Pten*^*wt/m3m4*^ and wildtype NSCs (*p* value = 0.020). **h** Quantification of branch intersection of neurites, showing consistently increased branch complexity in *Pten*^*m3m4/m3m4*^ NSCs at increasing distance from nucleus (*p value < 0.05; **p value < 0.01; ****p value < 0.0001)

To understand the nature of deficits in neuronal differentiation in *Pten*^*m3m4/m3m4*^ NSCs, we decided to monitor the respective immature and mature population of neurons over a longer course of differentiation (Fig. 4a–d). Consistent with our previous findings (Fig. 3), we found an increase in the number of Tuj1-positive cells in *Pten*^*m3m4/m3m4*^ NSCs at earlier differentiation timepoints, specifically 5 and 7 DIV (*p* values = 0.050 and 0.013, respectively). However, even with extended differentiation periods, *Pten*^*m3m4/m3m4*^ NSCs maintained an increased number of Tuj1-positive neurons compared to both *Pten*^*m3m4/wt*^ and *Pten*^*wt/wt*^ NSCs (Fig. 4a, b). This was correlated with significantly decreased numbers of NeuN-positive cells in *Pten*^*m3m4*^ mutant NSC cultures over 14 days of differentiation, where NeuN-positive cells were decreased in *Pten*^*m3m4/m3m4*^ versus wildtype at 7, 10, and 14 DIV (*p* values = < 0.0001, < 0.0001, and 0.0030, respectively; Fig. 4c, d). We observed that while *Pten* wildtype NSCs progressively differentiated into mature neurons over a time course of 14 days in culture, NSCs with *Pten*^*m3m4*^ mutations showed some NeuN-positive staining at day 7 in culture, but eventually failed to develop a robust population of NeuN-positive neurons. Cultures were terminated at 14 days of differentiation because we observed increased cell death at this timepoint. Consistent with these observations, we demonstrate that the ratio of NeuN-positive to Tuj1-positive cells increases in wildtype NSCs but decreases in homozygous mutant NSCs (*p* value = 0.0012; Fig. 4e). Moreover, the *Pten*^*m3m4/m3m4*^ NSCs exhibit an increase in both length and complexity of neurites compared to wildtype, a morphological phenotype indicative of immature status (Fig. 4f–h). There are significant increases in the average length of the longest neurite in both *Pten*^*wt/m3m4*^ (*p* value = 0.020) and *Pten*^*m3m4/m3m4*^ (*p* value = 0.0006) NSCs at 10 DIV relative to wildtype NSCs (Fig. 4g). These findings collectively suggest that neuronal differentiation of *Pten*^*m3m4/m3m4*^ NSCs is precocious yet stunted, resulting in a larger pool of immature neurons. *Pten*^*m3m4/m3m4*^ NSCs rapidly differentiate into immature neurons in greater number than wildtype NSCs, but the increased pool of immature neurons fail to reach maturity at the same rate as wildtype NSCs.

### Limited response in *Pten*^*m3m4/m3m4*^ NSCs to neuronal differentiation induction

**Fig. 5:**
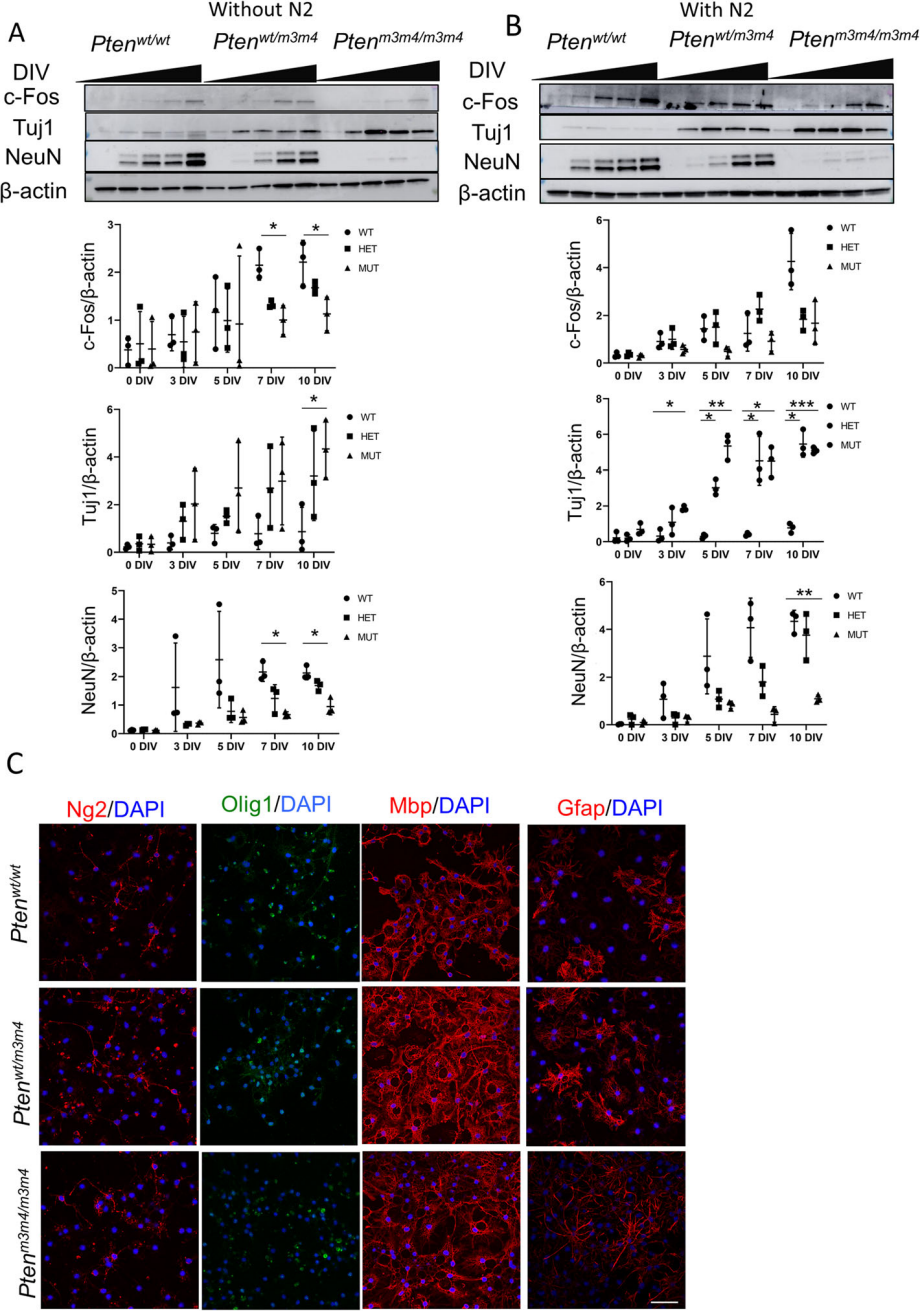
*Pten*^*m3m4*^ NSCs exhibit changes in neuronal activity and glia differentiation. **a** Western blot analysis (*n* = 3) assessing c-Fos, Tuj1, and NeuN expression in differentiated NSCs without specific lineage-driving growth factors (top panel) with quantification (bottom panel). Two-way ANOVA with Tukey-Kramer post hoc testing found significant decrease in c-Fos and NeuN in *Pten*^*m3m4/m3m4*^ versus wildtype at 7 DIV (*p* value = 0.025; 0.021) and 10 DIV (*p* value = 0.034; 0.014). A significant increase in Tuj1 between *Pten*^*m3m4/m3m4*^ and wildtype at 10 DIV was also observed (*p* value = 0.043). Two-way ANOVA with Tukey-Kramer post hoc testing found significant decrease in NeuN in *Pten*^*m3m4/m3m4*^ versus wildtype at 10 DIV (*p* value = 0.0066). Several significant increase in Tuj1 between *Pten*^*m3m4/m3m4*^ and wildtype at 3, 5, 7, and 10 DIV were also observed (*p* value =0.017; 0.0099; 0.025; 0.0002, respectively). **b** Western blot analysis (*n* = 3) shows c-Fos, Tuj1, and NeuN expression in differentiated NSCs treated with N2 supplement (top panel) with quantification (bottom panel). Densitometric quantification for specific protein expression is normalized to ß-actin. **c** Immunofluorescent staining at 10 DIV without growth factors for oligodendrocyte precursors cell (OPC), oligodendrocyte (OL), and myelination markers: Ng2 (red), Olig1 (green), Mbp (red), and Gfap (red), respectively (*p value < 0.05; **p value < 0.01; ***p value < 0.001; ****p value < 0.0001)

To investigate functional implications of a defect in maturation of *Pten*^*m3m4/m3m4*^ NSCs into mature neurons, we investigated their neuronal activity. We measured c-Fos expression, a proxy for neuronal activity, by Western blot in differentiated *Pten*^*m3m4/m3m4*^ NSCs. We found that c-Fos expression was decreased in differentiated *Pten*^*m3m4/m3m4*^ NSCs relative to both differentiated wildtype and *Pten*^*wt/m3m4*^ NSCs, suggesting that neuronal activity in culture correlates with the population of mature neurons (*p* value = 0.0022). This observation is supported by the trends in NeuN and Tuj1 expression, which show a significant decrease (*p* value = 0.0042) and increase (*p* value = 0.003) in differentiated *Pten*^*m3m4/m3m4*^ NSCs, respectively (Fig. 5a). Next, we sought to assess whether neuronal growth factor-driven NSC differentiation would alter the efficiency of *Pten*^*m3m4/m3m4*^ NSC differentiation into mature neurons. We found that addition of N2 supplement to NSCs cultures did, in fact, increase NeuN expression in mutant NSCs (assessed by visual inspection), suggesting an increased drive towards maturation in the population of NSCs (Fig. 5b, third panel); however, the *Pten*^*m3m4/m3m4*^ expression of NeuN was still significantly decreased compared to wildtype after 10 days of differentiation driven by growth factors (*p* value = 0.0066). The expression of c-Fos responded to N2 treatment, increasing in all genotype groups, though showing the lowest expression levels in the homozygous mutant NSCs (Fig. 5b). This was underscored by the finding of no-significant difference in NeuN expression across genotypes and timepoints of differentiation (*p* value = 0.095). Moreover, N2 treatment provoked a significant increase in Tuj1 expression in mutant NSCs (*p* value = 0.0007). These results underscore the finding that *Pten*^*m3m4/m3m4*^ NSCs prematurely arrest maturation because decreased neuronal activity is indicative of reduced mature neurons in cultures.

### Patterns of glial differentiation

Given the precocious yet abortive pattern of neuronal differentiation, and our previous work describing the white matter abnormalities and astrogliosis in the *Pten*^*m3m4*^ mouse [20, 22, 25], we were interested in any associated changes in oligodendrocyte (OL) lineage and astrocyte differentiation in *Pten*^*m3m4*^ NSC cultures. After 10 days of random differentiation, we stained for markers of OL and astrocyte differentiation: Ng2, Olig1, Mbp, and Gfap. We found no changes in Ng2, Olig1, and Mbp expression suggesting no differences in the number of oligodendrocyte precursor cells (OPCs) or OLs, respectively (Fig. 5c, d). Although we did not find any expression differences in OL makers, we found an increase in Gfap expression as well as morphological changes in astrocytes differentiated from *Pten*^*m3m4/m3m4*^ NSC, indicating astrogliosis (*p* value = *0.032*; Fig. 5c; Additional file 1: Figure S4A). These data highlight the effect that Pten disruption has on glial differentiation in addition to the effects on neuronal differentiation. The increase in astrocyte proliferation and change in morphology is striking.

### Activation of Creb correlates with differentiation

**Fig. 6:**
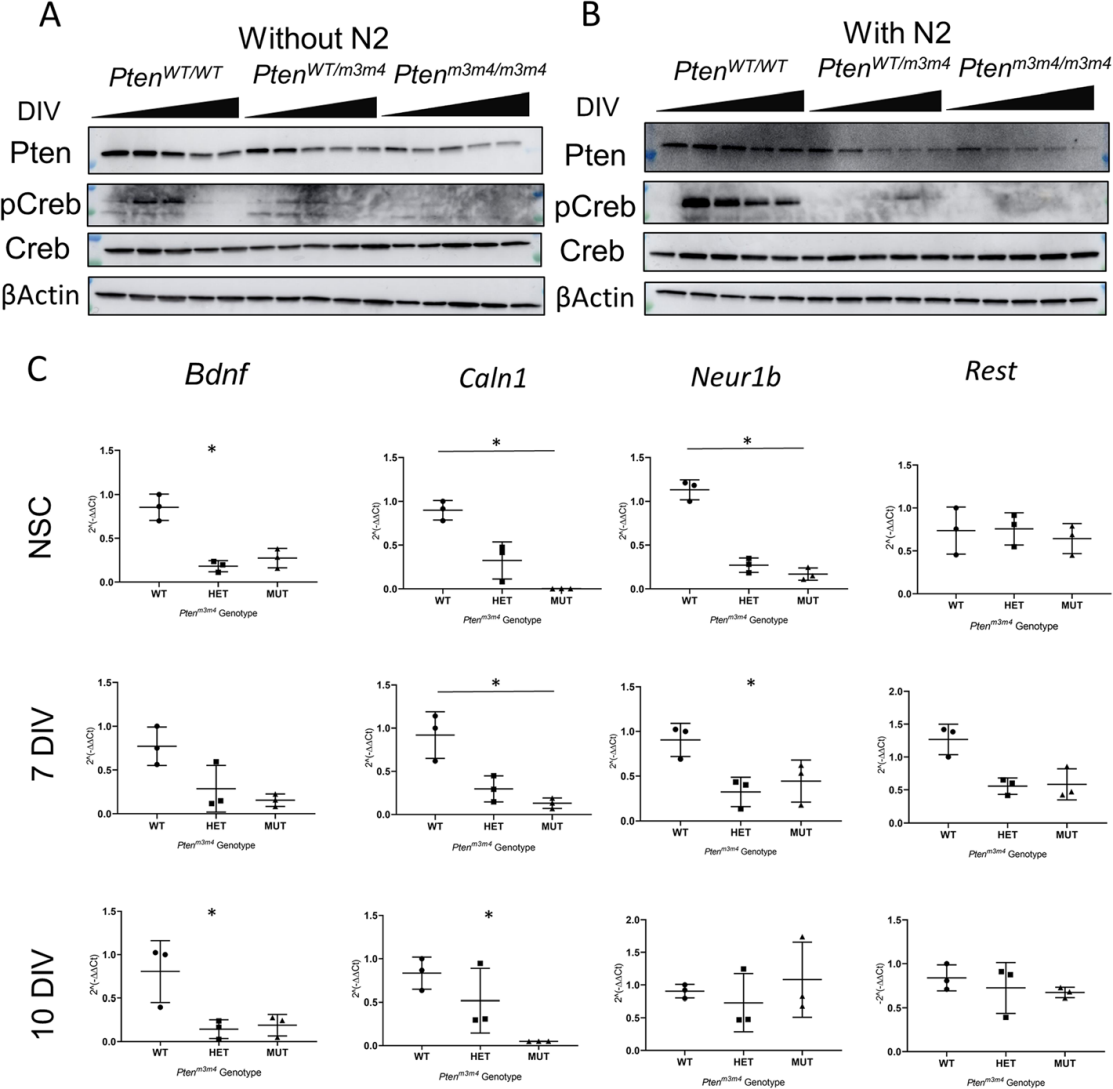
Changes in Creb activation in *Pten*^*m3m4*^ NSCs. **a** Western blot analysis of differentiated NSCs at 0, 3, 5, 7, and 10 DIV (depicted as rising black bar) without N2 growth factors. **b** Western blot analysis of differentiated NSCs at 0, 3, 5, 7, and 10 DIV (depicted as rising black bar) with N2 supplement (*n* = 3). **c** Transcript expression of Creb-regulated genes (*Bdnf*, *Caln1*, *Nerl1b*, and *Rest*) quantified by qRT-PCR at three timepoints: NSC, 7 DIV, and 10 DIV. For the NSC timepoint, Kruskal-Wallis testing found a significant difference in expression in *Bdnf* (*p* value = 0.025), *Caln1* (*p* value = 0.0036), and *Neur1b* (*p* value = 0.011) among genotype groups, where Dunn’s multiple comparison testing identified a significant difference between mutant and wildtype expression of Caln1 (*p* value = 0.022) and Neur1b (*p* value = 0.034). For the 7 DIV timepoint, Kruskal-Wallis test found a significant difference in expression in *Caln1* (*p* value = 0.011) and *Neur1b* (*p* value = 0.025) among genotype groups, where Dunn’s multiple comparison testing identified a significant difference between mutant and wildtype expression of Caln1 (*p* value = 0.034). For the 10 DIV timepoint, Kruskal-Wallis test found a significant difference in expression in *Bdnf* (*p* value = 0.025) and *Caln1* (*p* value = 0.025) among genotype groups. Three biological replicates (*n* = 3) were performed for each genotype group at each timepoint and relative transcript expression was measured in 2^−(∆∆Ct)^ (*p value < 0.05)

Previous work has demonstrated a correlation between Pten loss and Creb activation in NSCs [10]. Thus, we sought to examine whether the relationship between the m3m4 mutation in Pten and Creb activation in NSCs. As such, we differentiated *Pten*^*m3m4*^ NSCs with or without N2 supplement and then measured Creb phosphorylation at S133, a residue subject to dephosphorylation by Pten [10]. Surprisingly, we observed a decline in (almost absence of) Creb phosphorylation in both *Pten*^*wt/m3m4*^ and *Pten*^*m3m4/m3m4*^ NSCs independent of N2 supplement treatment (Fig. 6a, b). The N2 supplement treatment did slightly alter the pattern of Creb phosphorylation in wildtype NSCs, causing it to peak at day three, followed by a progressive decline, as opposed to peaking at day five followed by a steep decline in expression where differentiation was induced without N2 supplement (Fig. 6a, b). As part of a validation effort to confirm changes in Creb activation, we measured relative expression of transcripts known to be regulated by Creb (*Bdnf*, *Caln1*, *Neur1b*, and *Rest*) at three timepoints (0, 7, and 10 DIV). Consistent with the findings of the Western blot analysis, we observed significant decreases in the gene expression of Creb-regulated, neurodevelopmentally relevant targets. Moreover, we found the dysregulation occurred more broadly at the undifferentiated timepoint for NSCs, where three of four targets were significantly changed versus only 2 at differentiated timepoints. Interestingly, the dysregulation of Creb targets was not comprehensive. For instance, despite showing lower expression on average in the mutant NSC genotypes, *Rest* was not significantly decreased in expression compared to wildtype (Fig. 6c). Collectively, these results illustrate that changes in Creb activation are at least temporally associated with the observed deficits in differentiation in *Pten* mutant NSCs.

### Bdnf partially restores neuronal maturation in mutant NSCs

**Fig. 7:**
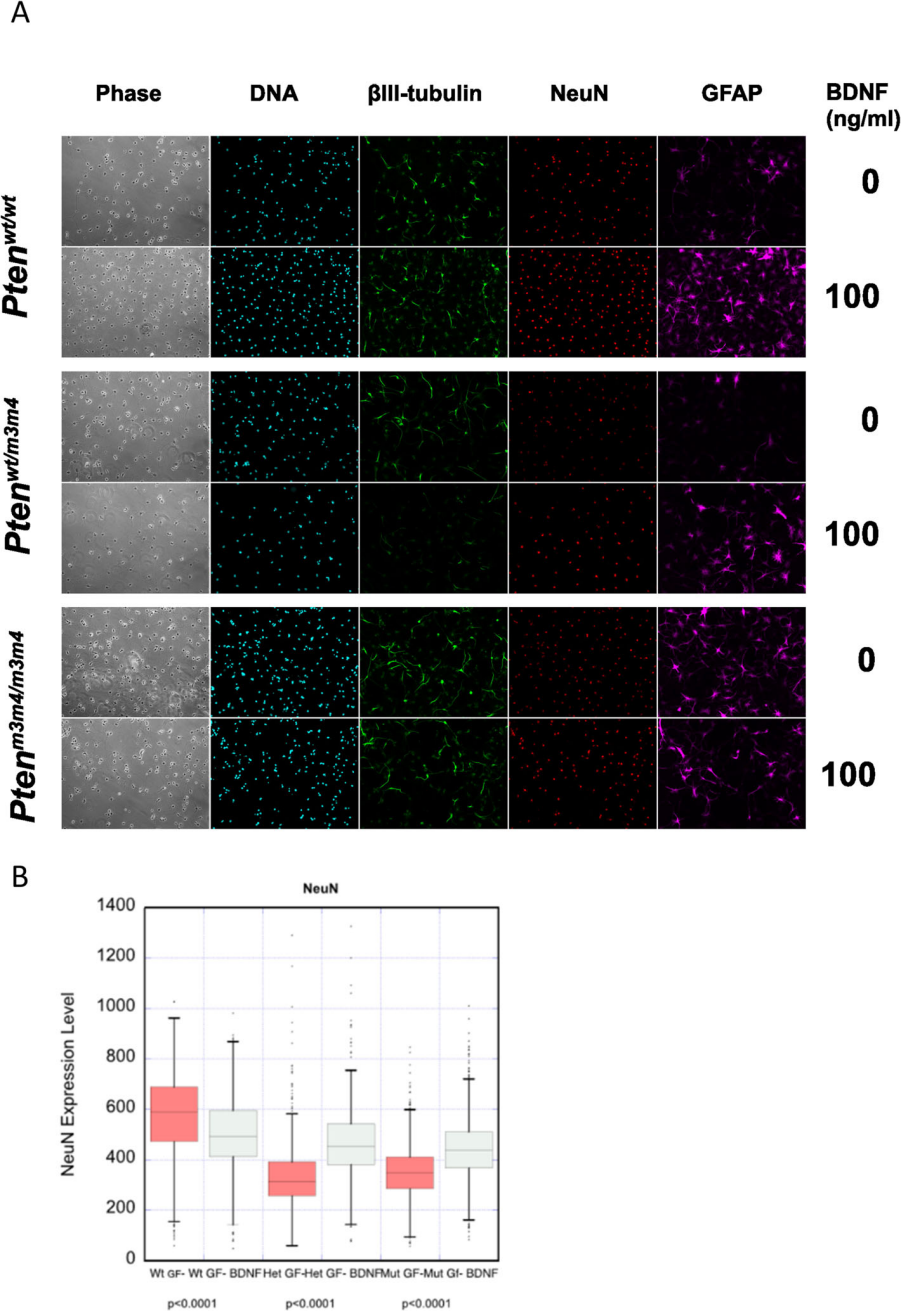
BDNF rescue of neuronal maturation in *Pten*^*m3m4*^ NSCs. **a** Immunofluorescent staining for Tuj1, NeuN, Gfap, and nuclear DNA with or without 100 ng/μL BDNF treatment. **b** Cell-based quantification of NeuN staining across all *Pten*^*m3m4*^ NSC genotypes

The finding of decreased Bdnf expression subsequent to the decrease in Creb activation may be related to the NeuN phenotype due to the well-described role of Bdnf in promoting neurogenesis. To assess whether Bdnf treatment can rescue the decrease in NeuN expression in the homozygous mutant NSCs, we treated *Pten*^*m3m4*^ NSCs with Bdnf and stained for Tuj1, NeuN, and Gfap (Fig. 7a). We found that that Bdnf treatment significantly increased NeuN expression in the heterozygous and homozygous mutant NSCs (*p* value < 0.0001); however, there rescue was only partial in effect not returning mutant NeuN to wildtype-like levels (Fig. 7b). These data illustrate that decreased Bdnf expression in *Pten*^*m3m4*^ NSCs is responsible, in part, for their stunted neuronal maturation.

## Discussion

Consistent with previous studies, our data show an increase in cellular proliferation and enhanced neurogenesis in NSCs with depleted Pten expression [14, 31–35]. In contrast, we show that a constitutive decrease in Pten expression, prominently in the nucleus, as observed in the *Pten*^*m3m4*^ model, is associated with stunted neuronal maturation, despite enhanced neurogenesis, after *Pten*^*m3m4*^ NSCs become immature yet post-mitotic neurons. The resulting accumulation of immature neurons is rapid and striking, where few mutant neurons progress to complete maturity, marked by NeuN expression (Figs. 3 and 4). The precocious yet abortive pattern of neuronal differentiation may be explained by the prolonged elevation in the expression of stem markers and subsequently the division/self-renewal capacity of *Pten*^*m3m4*^ mutant NSCs. Some of this differentiation pattern may also be due to the surprising lack of Creb activation in mutant NSCs, which also decrease Bdnf levels (Figs. 6 and 7). Although stunted maturation is evident only in the neuronal lineage, there appears to be some differential effects on glia: apparently unaffected OL differentiation and a clear increase in astrocyte proliferation and activation (Figs. 5 and 7). These findings are largely consistent with reported phenotypic observations in adult *Pten*^*m3m4*^ mice, where behavioral features reminiscent of high-functioning autism are observed, where morphological and physiological changes in neurons and astrocytes are observed without changes in cell numbers [25].

In our previous work, we characterized OPC differentiation isolated from the developing cortex of newborn *Pten*^*m3m4*^ mice. Interestingly, we found precocious, enhanced proliferation and increased myelin production; however, the myelin deposition observed is abnormal, generating bleb-like structures in the OLs, which often occur adjacent to, rather than, circumferentially, engulfing axons. In fact, we found that myelin thickness significantly decreased around mutant axons, which showed increased caliber [22]. It is possible that our study failed to observe a white matter phenotype because of the differing cellular contexts of the two studies. The OPC differentiation and dysmyelination in the *Pten*^*m3m4*^ model [22, 25] was observed in OPCs isolated from P2 cortices versus the NSCs from this study isolated from P20 DG. However, it remains unclear why the m3m4 mutation did not generate a white matter phenotype in NSCs in vitro. One can speculate cell-cell interactions are critically important in a 3-dimensional context in enacting the white matter phenotype.

In contrast to OL differentiation, astrocyte differentiation observed in mutant NSCs faithfully recapitulates the astrogliosis phenotype observed in the *Pten*^*m3m4/m3m4*^ brain [25]. Importantly, the NSC differentiation data show that the m3m4 mutation can affect both neuronal and glial lineages and in a fashion that is likely specific to each lineage.

The deficits in neuronal maturation in *Pten*^*m3m4*^ NSCs may, in part, be explained by surprising changes in Creb activation in *Pten*^*m3m4*^ NSCs, and its subsequent effect on Bdnf expression. A previous study demonstrated that the protein phosphatase activity of PTEN on S133 of CREB is critical in the regulation of neuronal differentiation, where Pten knockdown or disruption of the protein phosphatase activity of Pten increases the population of Tuj1-positive neurons during differentiation [10]. Our findings are consistent in that Pten disruption results in an increased pool of Tuj1-positive neurons; however, our data on the phosphorylation of Creb is divergent. We see a paradoxical reduction of Creb phosphorylation relative to Pten expression in the nucleus (Fig. 6a, b). It is possible that the decline in the nuclear localization of Pten would induce another phosphatase to decrease Creb phosphorylation or that the m3m4 Pten mutant may regulate cell signaling in a fashion that indirectly prevents increased Creb phosphorylation or that rampantly increased Pi3k/Akt signaling induces a feedback mechanism that reverses Creb activation. What is clear is that the lack of Creb activation associated with a decrease in Bdnf expression and that Bdnf treatment can partially rescue neuronal maturation in *Pten*^*m3m4*^ NSCs. This highlights the need for further research into the effects of Creb phosphorylation on neuronal maturation and the non-canonical roles of Pten in regulating this process.

An important distinction between our data and Lyu and colleagues’ data [10] is that we quantitatively examined differentiation of mature neurons (i.e., the NeuN-positive and Syn-positive populations), whereas their primary quantitative measure of neuronal differentiation was Tuj1-positive staining, a marker of post-mitotic yet immature neurons. This precludes a direct comparison with our findings. The mature neuron population in their model may be different, and this potential difference may explain the difference in the Creb activation data. Overall, our data suggest that the role of Pten in neuronal differentiation can be critically yet subtly tuned by different mutations, especially when comparing a cell-specific, conditional knockout model to a constitutive model of missense mutation. This implicates many of the non-canonical functions of Pten, which generally occur in the nucleus. Additionally, our data are consistent with the Lyu and colleagues’ study in that we find an increase in neurite length in differentiated mutant NSCs, but diverge in that we find changes in glia, specifically astrocytes differentiation where they did not.

In this study, we have identified a more complex, specified role for *Pten* in neurogenesis and neuronal maturation. We illustrate that *Pten* is more than a negative regulator of neurogenesis and neuronal maturation, and its participation in these processes is likely modulated by mutation type. A wealth of research has established that Pten is an important regulator of normal neuronal development, checking proliferation, promoting differentiation, and balancing maturation [36–38]. Our study expands upon these findings, while illustrating the nuanced effects that non-canonical Pten functions, especially in the nucleus, likely have on neuronal differentiation.

## Limitations

Although NSCs are an excellent model to study ASD pathogenesis, and Pten makes for a straightforward genetic model of ASD, our study is limited in scope in that only one *Pten* genotype was investigated, albeit, this genotype represents the common endpoint of a major subset of non-canonical signaling. Moreover, this endpoint is largely representative of a group of different patient mutations (i.e., those that disrupt Pten nuclear localization and stability and occur at residues distant from the catalytic motif). Despite the *Pten*^*m3m4*^ model’s faithful recapitulation of the clinical phenotypes of macrocephalic ASD as a component of PHTS, it is difficult to parse which phenotypes are attributable to the changes in localization versus the changes in expression of Pten versus changes in function specific to the m3m4 mutation itself. Ultimately, these questions can only be answered by comprehensively modeling patient mutations in iPSCs and animal models.

## Conclusion

Ultimately, we show that m3m4 mutation of Pten decreases nuclear localization and global expression of Pten in NSCs, a pattern that is exacerbated by differentiation (Fig. 2). Next, we demonstrate persistent stemness or a proclivity for progenitor status in *Pten*^*m3m4/m3m4*^ NSCs, which is marked by maintained upregulation of c-Myc (Fig. 1a, b). Subsequently, *Pten*^*m3m4/m3m4*^ NSCs exhibit increased proliferation (Fig 1c–e). Consistent with the stemness and proliferation results, we observe an accumulation of immature neurons from differentiated *Pten*^*m3m4/m3m4*^ NSCs that fail to reach maturity (Figs. 3 and 4). The deficits in neuronal differentiation are accompanied by deficits in glial differentiation, specifically astrogliosis (Fig. 5c). To simply illustrate the pattern of neurogenesis and gliogenesis markers over differentiation time, we have constructed a summary schematic (Additional file 1: Figure S5). Changes in differentiation may be partially mediated by changes in Creb activation in *Pten*^*m3m4/m3m4*^ NSCs (Fig. 6). This is underscored by the ability of Creb-regulated Bdnf to partially rescue neuronal maturation in mutant NSCs. Because we have already shown that the *Pten*^*m3m4/m3m4*^ neural transcriptome affects genes responsible for idiopathic ASD [39], this current study expands our knowledge concerning PTEN-ASD pathogenesis, and possibly all ASD, defining a critical role for non-canonical, likely nuclear, Pten function in neurogenesis, gliogenesis, and neuronal maturation.

## Supplementary information

**Additional file 1:** Supplementary Information. Decreased nuclear Pten in neural stem cells contributes to deficits in neuronal maturation. **Figure S1.** Neural stem cells (NSCs) derived from *Pten*^*m3m4*^ mice have higher stemness characteristics. **a** NSCs derived from dentate gyrus (DG) of wildtype (left, *n*=3), *Pten*^*wt/m3m4*^ (center, *n*=4), and *Pten*^*m3m4/m3m4*^ (right, *n*=4) mice had similar morphology after 1 day and 5 days (*n*=3) of *in vitro* growth in cell cultures containing growth factors (EGF and FGF2). Images shown in 20X. *Pten*^*m3m4*^ mutant NSCs showed a lack of contact inhibition (white arrow). **b** Quantification of c-Myc staining in Figure 1a, showing a significant increase in c-Myc expression in the homozygous mutant relative to wildtype (*p*-value = 0.051). **c** Quantification of c-Myc expression at 5 DIV finding a significant increase in expression for *Pten*^*m3m4/m3m4*^ NSCs compared to wildtype (*p*-value = 0.035) and *Pten*^*wt/m3m4*^ (*p*-value = 0.0042) NSCs (*p*-value = 0.035) as assessed by one-way ANOVA with Tukey-Kramer post hoc testing. **d** Quantification of Ki67 immunofluorescence at 0 and 3 DIV, finding a significant difference in Ki67+ nuclei between *Pten*^*m3m4/m3m4*^ and wildtype NSC at 0 (*p*-value = 0.036) and 3 (*p*-value < 0.0001) DIV as assessed by one-way ANOVA with Tukey-Kramer post hoc testing. There was also a significant increase in Ki67+ nuclei between *Pten*^*wt/m3m4*^ and wildtype NSCs at 3 DIV (*p*-value = 0.0039) as assessed by one-way ANOVA with Tukey-Kramer post hoc testing **e** Western blot analysis of undifferentiated NSCs (*n* = 3), showing increased Ccnd1 (*p*-value = 0.014) and P27kip1 (*p*-value = 0.039) expression in *Pten*^*m3m4/m3m4*^ NSCs compared to wildtype NSCs as assessed by ANOVA with Tukey-Kramer post hoc testing. A significant difference in expression of Ccnd1 between *Pten*^*m3m4/m3m4*^ and *Pten*^*wt/m3m4*^ NSCs (*p*-value = 0.011) was also found as assessed by ANOVA with Tukey-Kramer post hoc testing (*p-value < 0.05; **p-value < 0.01; ****p-value < 0.0001). **Figure S2.** NSCs with *Pten*^*m3m4*^ mutations show decreased nuclear and global Pten levels. **a** Densitometry quantification of ratio of Pten expression normalized to Gapdh in *Pten*^*m3m4/m3m4*^ NSCs vs *Pten*^*wt/wt*^ NSCs at 1 DIV (*p*-value = 0.0042). **b** Densitometric quantification of Western blot analyses of Pten expression normalized to Gapdh over 5 days of random differentiation. Two-way ANOVA testing found differences in Pten expression (*p*-value = 0.021) and between the time points (*p*-value = 0.036), where Pten expression explains 38% of the variance and time in culture explains 15%. No interaction was found between factors (Pten expression and DIV) was found. **c** Densitometric quantification of Western blot analysis of Pten, p110α (Pi3k), p-mTor, and p-Akt expression in *Pten*^*m3m4*^ NSCs. Kruskal-Wallis testing, accompanied by Dunn’s multiple comparison testing, found a significant decrease in Pten (*p*-value = 0.034) and a significant increase in p-mTOR (*p*-value = 0.034) and p-Akt (*p*-value = 0.034) in *Pten*^*m3m4/m3m4*^ versus wildtype NSCs. **Figure S3.** Synapthophysin staining on *Pten*^*m3m4*^ NSCs. **a** High magnification representative image of Syn+ (green) cells at seven DIV with DAPI (blue). Scale bar = 10 μm. **b** Top panel: Low magnification representative image of Syn+ (green) cells at 10 DIV with DAPI (blue). Bottom panel: High magnification of circled image in top panel. Scale bar = 20 μm. **c** Quantification percent Syn+ cells normalized to DAPI expression. Only significant difference between wildtype Syn expression at seven and 10 DIV (*p*-value < 0.05). Scale bar = 20 μm. **Figure S4.** Quantitative view of gliagenesis in *Pten*^*m3m4*^ NSCs. **a** Quantification of gliagenesis staining data in Figure 5, including Ng2, Olig1, Mbp, and Gfap. Gfap shows a significant difference in expression between wildtype and homozygous mutant by integrated density or stain area normalized to cell number (*p*-value = 0.032 and 0.017, respectively). **b** Gfap staining trace analysis followed by stain area and perimeter calculations.

## Data Availability

The datasets for this study are available from the corresponding author on reasonable request.

## Abbreviations

ASD: Autism spectrum disorder
PTEN: Phosphatase and tensin homolog on chromosome ten
DG: Dentate gyrus
NSC: Neural stem cells
OPC: Oligodendrocyte progenitor cell
OL: Oligodendrocyte
