# Supplementary material for: Decreased nuclear Pten in neural stem cells contributes to deficits in neuronal maturation
Source: Mol Autism. 2020 Jun 1;11:43. doi: 10.1186/s13229-020-00337-2 (PMC7268763; doi:10.1186/s13229-020-00337-2)
Supplement: Supplementary file 1 — Additional file 1: Supplementary Information. Decreased nuclear Pten in neural stem cells contributes to deficits in neuronal maturation. Figure S1. Neural stem cells (NSCs) derived from Ptenm3m4 mice have higher stemness characteristics. a NSCs derived from dentate gyrus (DG) of wildtype (left, n=3), Ptenwt/m3m4 (center, n=4), and Ptenm3m4/m3m4 (right, n=4) mice had similar morphology after 1 day and 5 days (n=3) of in vitro growth in cell cultures containing growth factors (EGF and FGF2). Images shown in 20X. Ptenm3m4 mutant NSCs showed a lack of contact inhibition (white arrow). b Quantification of c-Myc staining in Figure 1a, showing a significant increase in c-Myc expression in the homozygous mutant relative to wildtype (p-value = 0.051). c Quantification of c-Myc expression at 5 DIV finding a significant increase in expression for Ptenm3m4/m3m4 NSCs compared to wildtype (p-value = 0.035) and Ptenwt/m3m4 (p-value = 0.0042) NSCs (p-value = 0.035) as assessed by one-way ANOVA with Tukey-Kramer post hoc testing. d Quantification of Ki67 immunofluorescence at 0 and 3 DIV, finding a significant difference in Ki67+ nuclei between Ptenm3m4/m3m4 and wildtype NSC at 0 (p-value = 0.036) and 3 (p-value < 0.0001) DIV as assessed by one-way ANOVA with Tukey-Kramer post hoc testing. There was also a significant increase in Ki67+ nuclei between Ptenwt/m3m4 and wildtype NSCs at 3 DIV (p-value = 0.0039) as assessed by one-way ANOVA with Tukey-Kramer post hoc testing e Western blot analysis of undifferentiated NSCs (n = 3), showing increased Ccnd1 (p-value = 0.014) and P27kip1 (p-value = 0.039) expression in Ptenm3m4/m3m4 NSCs compared to wildtype NSCs as assessed by ANOVA with Tukey-Kramer post hoc testing. A significant difference in expression of Ccnd1 between Ptenm3m4/m3m4 and Ptenwt/m3m4 NSCs (p-value = 0.011) was also found as assessed by ANOVA with Tukey-Kramer post hoc testing (*p-value < 0.05; **p-value < 0.01; ****p-value < 0.0001). Figure S2. NSCs with Ptenm3m4 mutati [file 13229_2020_337_MOESM1_ESM.docx]

**Additional File 1: Supplementary Information**

**Decreased nuclear Pten in neural stem cells contributes to deficits in neuronal maturation**

Shin Chung Kang^1^, Ritika Jaini^1,2,3^, Masahiro Hitomi^1,2^, Hyunpil Lee^1^, Nick Sarn^1,5^, Stetson Thacker^1,2^, Charis Eng^1-5^*

^1^Genomic Medicine Institute, Lerner Research Institute, Cleveland Clinic, Cleveland, OH, 44195, USA

^2^Cleveland Clinic Lerner College of Medicine, Case Western Reserve University, Cleveland, OH, 44195, USA

^3^Case Comprehensive Cancer Center, Case Western Reserve University School of Medicine, Cleveland, OH, 44106, USA

^4^Taussig Cancer Institute, Cleveland Clinic, Cleveland, OH, 44195, USA

^5^Department of Genetics and Genome Sciences, Case Western Reserve University School of Medicine, Cleveland, OH, 44106, USA

*Correspondence: [engc@ccf.org](mailto:engc@ccf.org); Genomic Medicine Institute, Lerner Research Institute, Cleveland Clinic 9500 Euclid Avenue, Cleveland, OH 44195; Tel: (216) 444-3900

Authors’ contact information: Shin Chung Kang ([kangs3@ccf.org](mailto:kangs3@ccf.org)); Ritika Jaini ([jainir@ccf.org](mailto:jainir@ccf.org)); Stetson Thacker ([thackes@ccf.org](mailto:thackes@ccf.org)); Masahiro Hitomi ([hitomim@ccf.org](mailto:hitomim@ccf.org)); Hyunpil Lee ([leeh6@ccf.org](mailto:leeh6@ccf.org)); Nick Sarn ([sarnn@ccf.org](mailto:sarnn@ccf.org)); Charis Eng ([engc@ccf.org](mailto:engc@ccf.org))


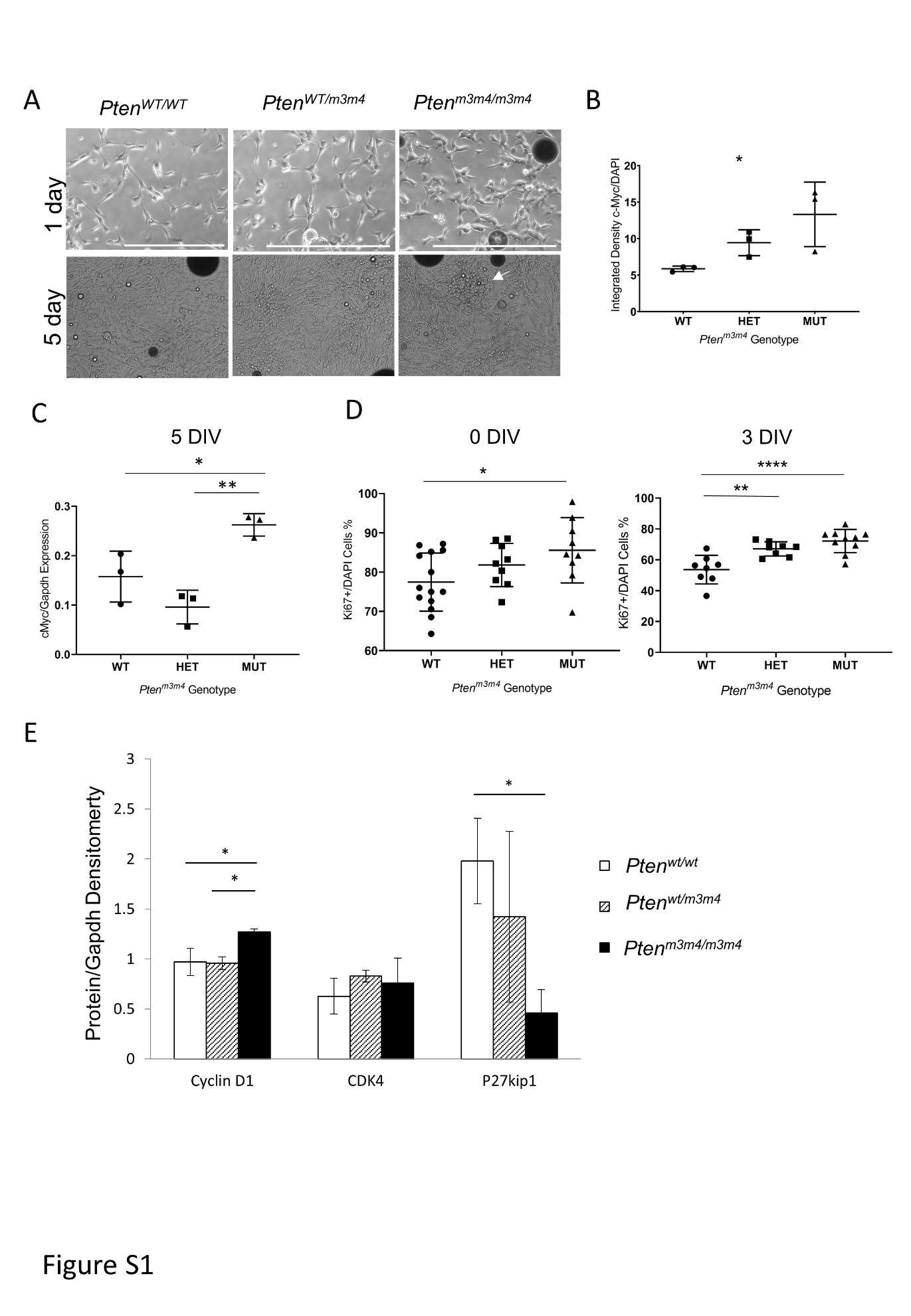


**Supplemental Figure S1.** Neural stem cells (NSCs) derived from *Pten^m3m4^* mice have higher stemness characteristics. **a** NSCs derived from dentate gyrus (DG) of wildtype (left, n=3), *Pten^wt/m3m4^* (center, n=4), and *Pten^m3m4/m3m4^* (right, n=4) mice had similar morphology after 1 day and 5 days (n=3) of *in vitro* growth in cell cultures containing growth factors (EGF and FGF2). Images shown in 20X. *Pten^m3m4^* mutant NSCs showed a lack of contact inhibition (white arrow). **b** Quantification of c-Myc staining in Figure 1a, showing a significant increase in c-Myc expression in the homozygous mutant relative to wildtype (p-value = 0.051). **c** Quantification of c-Myc expression at 5 DIV finding a significant increase in expression for *Pten^m3m4/m3m4^* NSCs compared to wildtype (p-value = 0.035) and *Pten^wt/m3m4^* (p-value = 0.0042) NSCs (p-value = 0.035) as assessed by one-way ANOVA with Tukey-Kramer post hoc testing. **d** Quantification of Ki67 immunofluorescence at 0 and 3 DIV, finding a significant difference in Ki67+ nuclei between *Pten^m3m4/m3m4^* and wildtype NSC at 0 (p-value = 0.036) and 3 (p-value < 0.0001) DIV as assessed by one-way ANOVA with Tukey-Kramer post hoc testing. There was also a significant increase in Ki67+ nuclei between *Pten^wt/m3m4^* and wildtype NSCs at 3 DIV (p-value = 0.0039) as assessed by one-way ANOVA with Tukey-Kramer post hoc testing **e** Western blot analysis of undifferentiated NSCs (n = 3), showing increased Ccnd1 (p-value = 0.014) and P27kip1 (p-value = 0.039) expression in *Pten^m3m4/m3m4^* NSCs compared to wildtype NSCs as assessed by ANOVA with Tukey-Kramer post hoc testing. A significant difference in expression of Ccnd1 between *Pten^m3m4/m3m4^* and *Pten^wt/m3m4^* NSCs (p-value = 0.011) was also found as assessed by ANOVA with Tukey-Kramer post hoc testing (*p-value < 0.05; **p-value < 0.01; ****p-value < 0.0001).


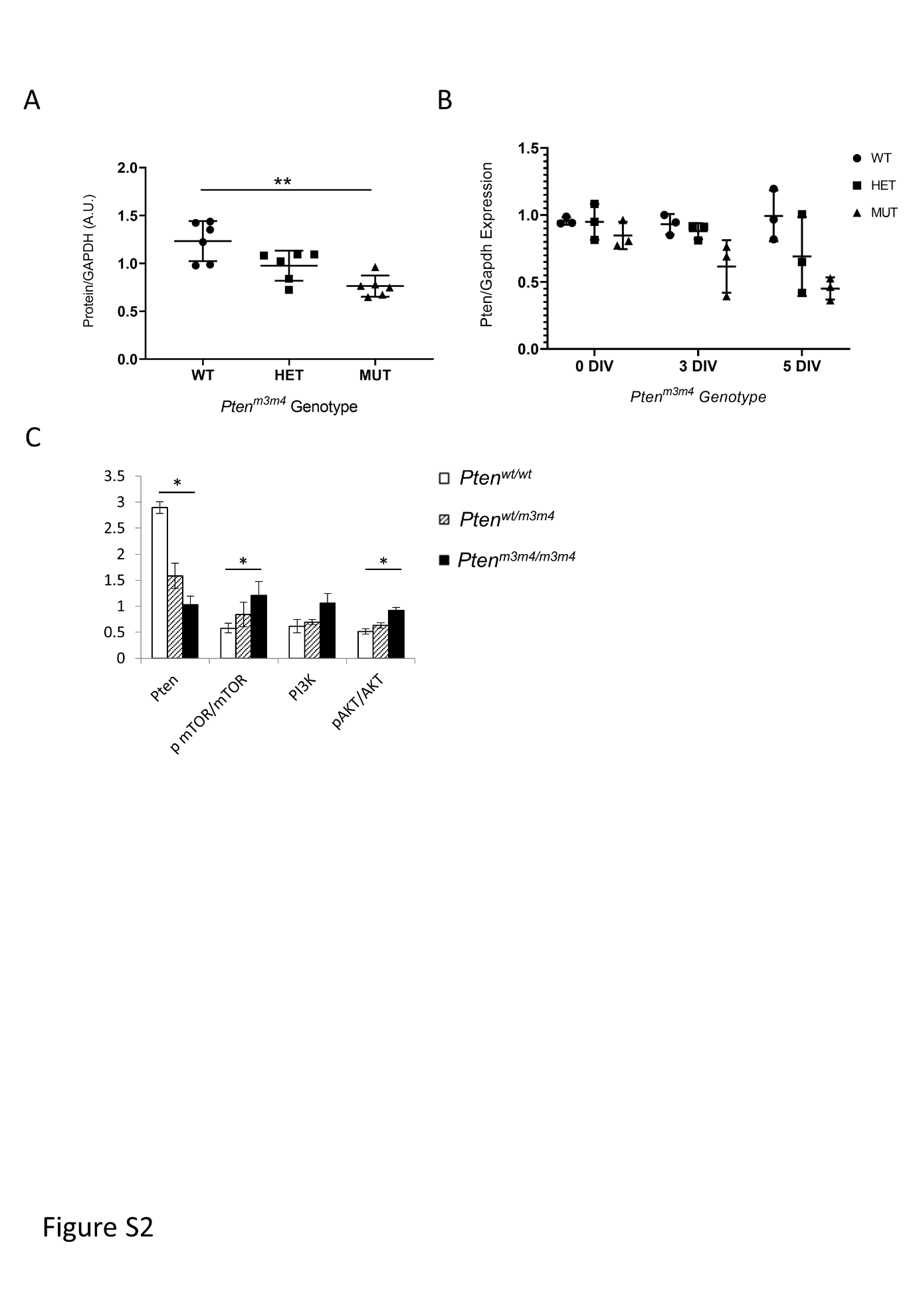


**Supplemental Figure S2.** NSCs with *Pten^m3m4^* mutations show decreased nuclear and global Pten levels. **a** Densitometry quantification of ratio of Pten expression normalized to Gapdh in *Pten^m3m4/m3m4^* NSCs vs *Pten^wt/wt^* NSCs at 1 DIV (p-value = 0.0042). **b** Densitometric quantification of Western blot analyses of Pten expression normalized to Gapdh over 5 days of random differentiation. Two-way ANOVA testing found differences in Pten expression (p-value = 0.021) and between the time points (p-value = 0.036), where Pten expression explains 38% of the variance and time in culture explains 15%. No interaction was found between factors (Pten expression and DIV) was found. **c** Densitometric quantification of Western blot analysis of Pten, p110α (Pi3k), p-mTor, and p-Akt expression in *Pten^m3m4^* NSCs. Kruskal-Wallis testing, accompanied by Dunn’s multiple comparison testing, found a significant decrease in Pten (p-value = 0.034) and a significant increase in p-mTOR (p-value = 0.034) and p-Akt (p-value = 0.034) in *Pten^m3m4/m3m4^* versus wildtype NSCs.


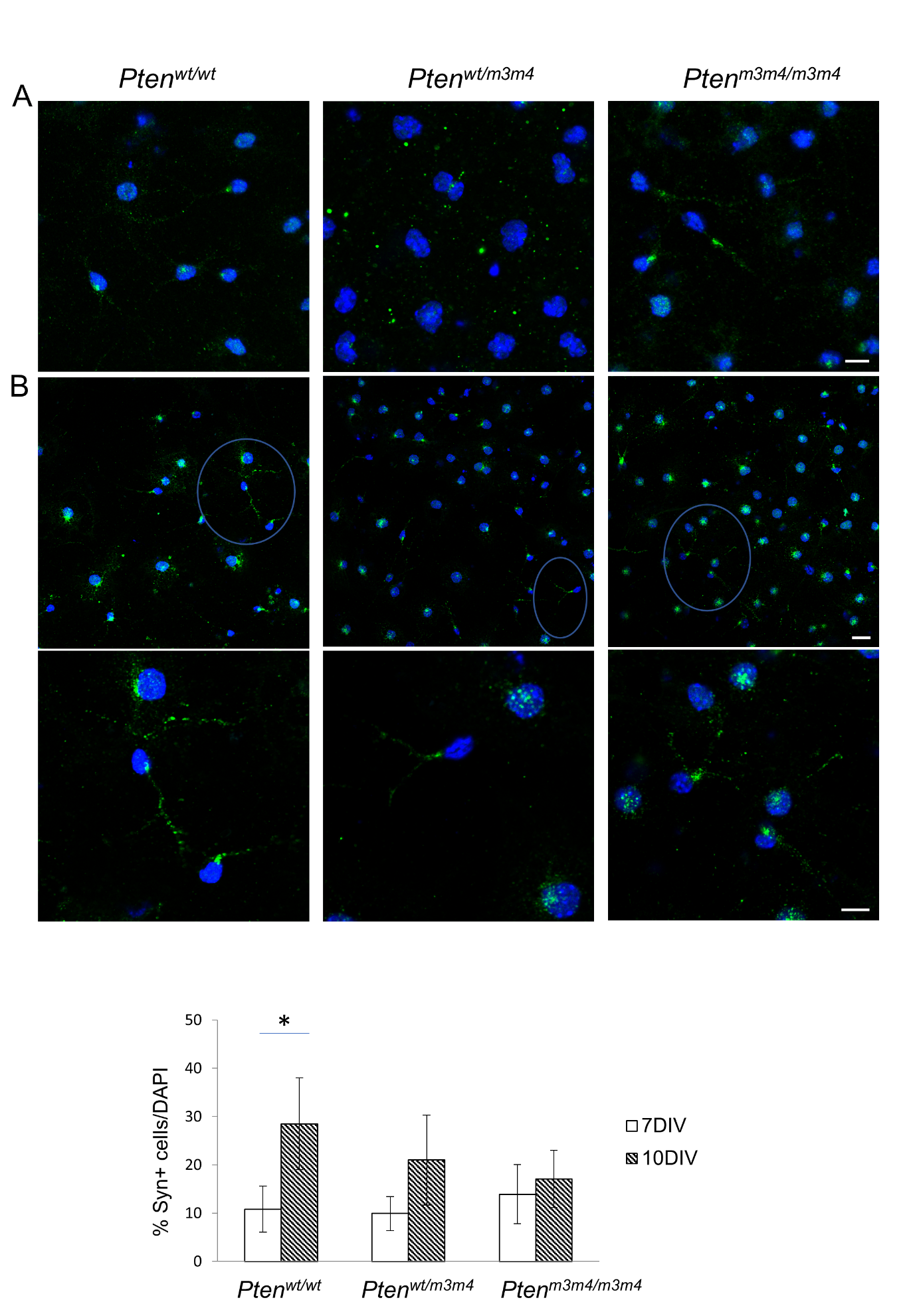


**Supplemental Figure S3.** Synapthophysin staining on *Pten^m3m4^* NSCs. **a** High magnification representative image of Syn+ (green) cells at seven DIV with DAPI (blue). Scale bar = 10 μm. **b** Top panel: Low magnification representative image of Syn+ (green) cells at 10 DIV with DAPI (blue). Bottom panel: High magnification of circled image in top panel. Scale bar = 20 μm. **c** Quantification percent Syn+ cells normalized to DAPI expression. Only significant difference between wildtype Syn expression at seven and 10 DIV (p-value < 0.05). Scale bar = 20 μm.


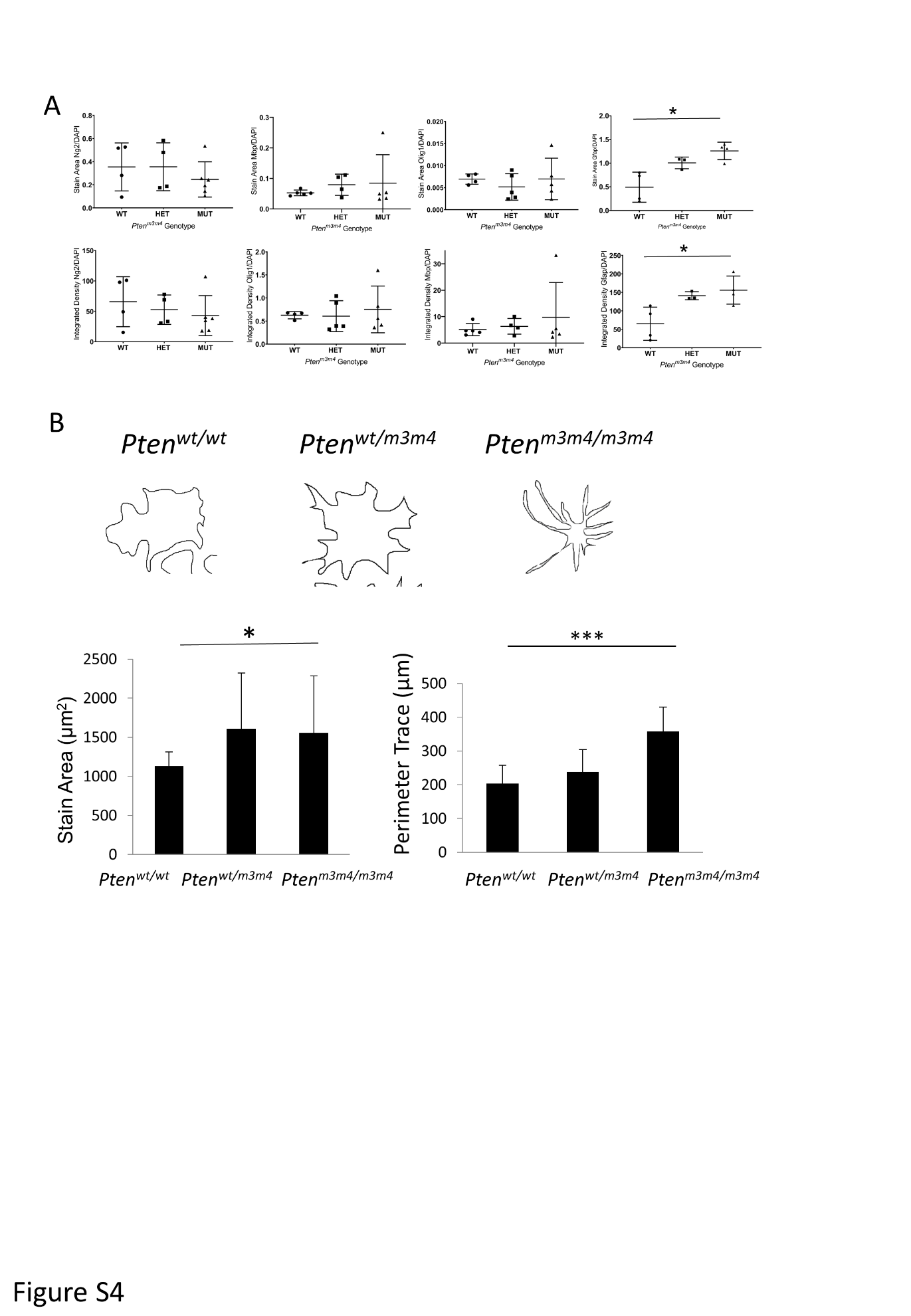


**Supplemental Figure S4.** Quantitative view of gliagenesis in *Pten^m3m4^* NSCs. **a** Quantification of gliagenesis staining data in Figure 5, including Ng2, Olig1, Mbp, and Gfap. Gfap shows a significant difference in expression between wildtype and homozygous mutant by integrated density or stain area normalized to cell number (p-value = 0.032 and 0.017, respectively). **b** Gfap staining trace analysis followed by stain area and perimeter calculations.
